# Genetic Characterization of Highly Pathogenic Avian Influenza (H5N8) Virus from Domestic Ducks, England, November 2014

**DOI:** 10.3201/eid2105.141954

**Published:** 2015-05

**Authors:** Amanda Hanna, Jill Banks, Denise A. Marston, Richard J. Ellis, Sharon M. Brookes, Ian H. Brown

**Affiliations:** Animal and Plant Health Agency, Addlestone, UK

**Keywords:** Highly pathogenic avian influenza, HPAI, H5N8, influenza, viruses, poultry, ducks, zoonoses, molecular epidemiology, England, China, Japan, South Korea, Germany, the Netherlands

## Abstract

Genetic sequences of a highly pathogenic avian influenza (H5N8) virus in England have high homology to those detected in mainland Europe and Asia during 2014. Genetic characterization suggests this virus is an avian-adapted virus without specific affinity for zoonoses. Spatio-temporal detections of H5N8 imply a role for wild birds in virus spread.

Aquatic birds are considered to be the natural reservoir of low pathogenicity avian influenza viruses of subtypes H1–H16; in these birds, including ducks, they generally do not cause clinical signs (*1*). In contrast, highly pathogenic avian influenza (HPAI) viruses of certain H5 and H7 strains cause high death rates in poultry with substantial economic losses and are thought to be derived from low pathogenicity avian influenza viruses of wild bird origin. China, Japan, and South Korea have reported outbreaks of highly related HPAI (H5N8) virus in poultry and migratory birds since early 2014 (*2*,*3*). Germany and the Netherlands have reported outbreaks among poultry with closely related H5N8 viruses in turkey, chicken, and duck farms since early November 2014. On November 16, 2014, an outbreak of HPAI (H5N8) virus was confirmed on a duck breeding farm in East Yorkshire, England, UK (*4*). The premises contained 6,000 breeding ducks ≈60 weeks of age housed in 3 sheds; the ducks showed only mild clinical signs of illness.

## The Study

Thirty-seven of 120 individual swab samples and 5 of 12 tissue pools (Technical Appendix) were confirmed positive for avian influenza virus by identification of the matrix (M) gene and H5 real-time reverse transcription PCR (rRT-PCR) as described (*5*). A highly pathogenic pathotype was confirmed by RT-PCR, then Sanger sequencing of the hemagglutinin (HA) gene as described (*5*). This process showed the polybasic cleavage site PLRERRRKR/GLF from multiple bird swab and tissue samples from each shed. HPAI virus was isolated in 9- to10-day-old specific pathogen–free embryonated hens’ eggs after incubating 2 days as described (*6*). A primary isolate, A/duck/England/36254/2014 (dkEng14), was obtained after egg inoculation with pooled intestinal samples from 1 shed and was typed as H5N8 by using standard protocols (*6*). We obtained full genome sequence from dkEng14 by next-generation sequencing (Technical Appendix). Full genome sequences of 2 more isolates, A/duck/England/36038/2014 and A/duck/England/36226/2014, derived from pooled cloacal swabs and pooled viscera from 2 different sheds, respectively, were also assessed by next generation sequencing and analyzed. We entered genome sequences on the Global Initiative on Sharing All Influenza Data EpiFlu database (*7*) under accession nos. EPI547670– EPI547677, EPI550848–EPI550849, and EPI558000–EPI558013. Initial maximum likelihood phylogenetic analysis of the HA gene was conducted as described (*5*) and the evolutionary analyses performed by using MEGA6 (*8*). 

**Figure 1:**
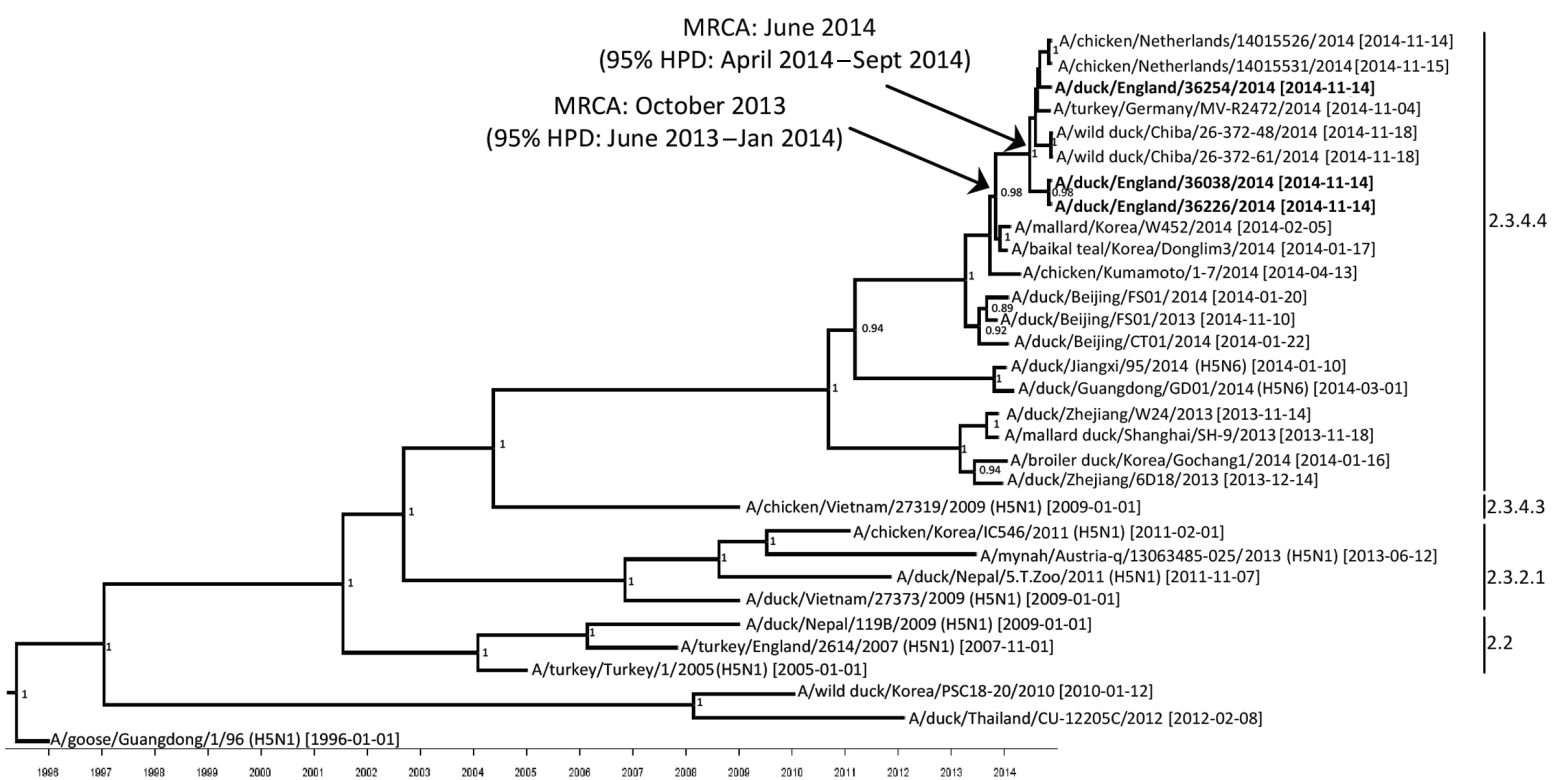
Maximum clade credibility tree of 31 H5 sequences derived from the hemagglutinin gene of avian influenza viruses (1,608 nt). Sampling dates and locations are included on the tip labels; where specific dates were unknown, ‘01’ was assigned. Node labels indicate significant posterior probabilities (>0.75). The dates for the most recent common ancestor (MRCA) of the currently circulating viruses circulating in Europe and Japan are indicated at the relevant nodes with 95% highest posterior density (HPD) levels. Sequences relate to H5N8 subtype unless otherwise noted. Sequences reported in this study are in bold.

Phylogenetic analysis showed 99.9%–100% similarity among the 3 duck isolates from the single premises in England and 99.4–100% similarity among HPAI (H5N8) sequences from China, Japan, South Korea, Germany, and the Netherlands; all belonged to Asian lineage H5 clade 2.3.4.4. Sequence comparisons based on 1,608 nt of the HA gene from each of the 3 duck isolates identified A/turkey/Germany/MV-R2472/2014 (H5N8) as the closest match, at 99.8% similarity. We implemented further phylogenetic analysis using Bayesian Markov chain Monte Carlo simulation in the BEAST package version 1.7 (*9*). The maximum clade credibility tree (Figure 1) had a similar topology to that observed for the maximum-likelihood tree. 

The time elapsed between the detection of the isolates in England and the most recent common ancestor (MRCA) of the H5N8 virus cluster from Europe and Japan was ≈5 months (95% highest posterior density [HPD] range 2.7–7.7 months; approximately June 2014). This cluster shares homology of the HA gene with viruses detected in South Korea in early 2014. The ancestor of the viruses from continental Europe, Japan, and Korea occurred ≈13 months (11–15.5 months, 95% HPD; approximately October 2013) before the detection of the English isolates. The separation between the H5N8 HA sequences from England is attributed to 2 nonsynonymous mutations coding for amino acid substitution S181P and H273Y (HA numbering based on the mature H5 protein). Position 181 is in close proximity to the receptor binding site and antigenic site Sb (*10*); however, the effect of this condition on antigenicity has not yet been defined. 

**Figure 2:**
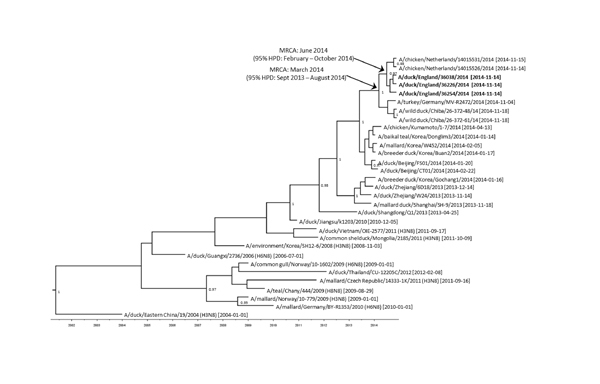
Maximum clade credibility tree of 31 N8 sequences derived from the neuraminidase gene of avian influenza viruses (1377 nt). Sampling dates and locations are included on the tip labels; where specific dates were unknown, ‘01’ was assigned. Node labels indicate significant posterior probabilities (>0.75). The dates for the most recent common ancestor (MRCA) are indicated at the relevant nodes with 95% highest posterior density (HPD) levels. Sequences relate to H5N8 subtype unless otherwise noted. Sequences reported in this study are in bold.

The HA sequence variation observed within 1 premises in which ducks were infected in England is potentially the consequence of virus adaptation within the flock subsequent to a single introduction. Full-genome comparison of the 3 viruses in England showed amino acid differences in the HA and NA genes only (Table 1). Further analyses with sequences from the NA gene indicated a similar most recent common ancestor (≈5 months) for the European lineage. However, each of the 3 virus sequences detected in England grouped together with those from the Netherlands (Figure 2).

**Table 1:** Genome sequence comparisons of 3 highly pathogenic avian influenza (H5N8) viruses from domestic ducks, England, November 2014*

Gene segment	Sequence		
	36038, 36226	36038, 36254	36226, 36254
PB2	0	1	1
PB1	0	0	0
PA	1	0	1
HA	0	2 (S181P, H273Y)	2 (S181P, H273Y)
NP	0	0	0
NA	1 (L363I)	4 (S164P, N166S, K186N, L363I)	3 (S164P, N166S, K186N)
MP	0	0	0
NS	0	0	0
*Sequences compared are A/duck/England/36038/2014, A/duck/England/36226/2014, and A/duck/England/36254/2014. Amino acid residue in parentheses indicate that the number of nucleotide differences correspond to a nonsynonomous change. PB2, polymerase basic 2; PB1, polymerase basic 1;PA, polymerase acidic; HA hemagglutinin; NP, nucleoprotein; NA, neuraminidase; MP, matrix; NS, nonstructural.			

To investigate the potential zoonotic affinity of dkEng14, sequence data was compared with the H5N1 genetic changes inventory at the US Centers for Disease Control and Prevention (*11*) to identify single or collective mutations that might influence viral phenotypic characteristics of importance and may indicate adaptation to mammalian species or alter susceptibility to antiviral drugs. A total of 8 point mutations among 114 of interest were identified (Table 2). In addition to the polybasic (PB) cleavage site in the HA protein, positions 133 and 156 each contained an alanine residue (H5 numbering relative to A/Vietnam/1203/2004), which is reportedly related to increased virus binding to α2,6 sialic acid human receptors. 

**Table 2:** Genetic mutations identified in highly pathogenic avian influenza (H5N8) virus isolate A/duck/England/36254/2014 that might result in phenotypic consequences, England, 2014*

Protein, amino acid position/motif	Phenotypic consequences†	
PB1-F2,		
N66S	Increased virulence, replication efficiency and antivirus response in mice
HA	
S133A	Increased psuedovirus binding to α2,6	
T156A	Increased virus binding to α2,6 and increased transmission in guinea pigs	
323–330 (R-X-R/K-R)	Polybasic cleavage motif sequence required for high pathogenicity	
M1	
N30D	Increased virulence in mice	
T215A	Increased virulence in mice	
M2	
S31N	Reduced susceptibility to amantadine and rimantadine antiviral drugs	
NS1	
P42S	Increased virulence in mice	
I101M	Increased virulence in mice	

*Phenotypic consequences may include an influence on viral phenotypic characteristics of importance, adaptation to mammalian species, or altered susceptibility to existing antiviral drugs. H5N1 numbering based on the mature HA protein relative to A/Vietnam/1203/2004. PB, polymerase basic protein; HA, hemagglutinin; M, matrix; NS, nonstructural.  †The mutation to the right of the amino acid position confers the phenotypic consequence described.

An asparagine that has been associated with reduced susceptibility to amantadine and rimantadine antiviral drugs was present at position 31 in the M2 protein, but we did not detect any signature motifs associated with resistance to antivirals targeting neuraminidase. Aspartic acid at position 30 and alanine at position 215 in the M1 protein together with serine at position 42 and methionine at position 101 in the nonstructural 1 protein were observed; all have been individually linked to increased virulence in mice. A serine residue at position 66 in the PB1-F2 protein was also observed. This mutation has also been linked to increased virulence in mice and studies in ducks showed a minor role in pathogenesis. Of interest is the lack of a deletion in the nonstructural 1 protein at amino acid positions 80–84 that is conserved among contemporary H5N1 viruses, possibly decreasing the zoonotic potential of the H5N8 virus. Two mutations in PB2, E627K, and K526R (*12*), described to be involved with mammalian host adaptation and increased replication in mammalian cells, were not observed. Eight mutations were identified in dkEng14; only 1 (T156A) of the 5 necessary mutations associated with high risk for zoonotic transmission were observed (*13*,*14*). The mutations identified in Table 2 are analogous to that of A/mallard duck/Korea/W452/2014 (H5N8), a virus that has been studied for its pathogenic and pandemic potential (*15*) and was found to result in impaired replication and inefficient contact transmission among ferrets.

## Conclusions

The genome of the H5N8 virus isolated in England suggests that it is still predominantly an avian-adapted virus, without any specific increased affinity for humans. Close genetic homology among the viral genes of the H5N8 viruses detected in England, the Netherlands, and Germany suggest they share a common ancestor with the recent H5N8 viruses isolated from wild ducks in Japan, a result of reassortment estimated to have occurred in June 2014. Reliable interpretation of the topology of the European and Japanese cluster cannot be made with these similar sequences. Phylogenetic analysis of sequences from more viruses will help to resolve these relationships. Detection of H5N8 (HPAI) viruses in 3 countries in Europe over a short time period in different poultry species without the establishment of clear epidemiologic links implicates a role for wild birds in spreading of viruses. The potential for further dissemination of HPAI (H5N8) viruses in Europe is a threat to poultry. Viral sequence analysis from new outbreaks is recommended to monitor virus evolution, understand risk pathways for introduction, and assess the emergence of mutations that may be relevant for veterinary and public health.

Technical Appendix Methods for genetic characterization of HPAI H5N8 virus among domestic ducks in England.
