# Supplementary material for: Genetic Characterization of Highly Pathogenic Avian Influenza (H5N8) Virus from Domestic Ducks, England, November 2014
Source: Emerg Infect Dis. 2015 May;21(5):879–82. doi: 10.3201/eid2105.141954 (PMC4412239; doi:10.3201/eid2105.141954)
Supplement: Technical Appendix — Methods for genetic characterization of HPAI H5N8 virus among domestic ducks in England. [file 14-1954-Techapp-s1.pdf]

Article DOI: http://dx.doi.org/10.3201/eid2105.141954

Genetic Characterization of Highly Pathogenic Avian Influenza (H5N8) Virus Among Domestic Ducks, England, November 2014

Technical Appendix: Methods for genetic characterization of HPAI H5N8 virus among domestic ducks in England

Methods

Sample Preparation

Twenty oropharyngeal swabs, 20 cloacal swabs and two carcasses were collected from each of the three sheds. Tissue samples collected from each carcass were pooled per shed; brain, lung and trachea, intestines and mixed viscera (liver, heart, spleen and kidney) for testing by rRT PCR. Individual swabs were pooled in groups of five per shed, together with the pooled tissues for virus isolation.

Next Generation Sequencing

Double stranded cDNA (cDNA synthesis system, Roche, UK) was generated from RNA according to the manufacturer's instructions. This was quantified using the fluorescent PicoGreen reagent and 1ng was used as a template for the preparation of the sequencing library (NexteraXT, Illumina, Cambridge, UK). Sequencing libraries were run on a MiSeq instrument (Illumina, Cambridge, UK) with 2x75 base paired end reads.

Phylogenetic Sequence Analysis

Phylogenetic analysis was implemented using Bayesian Markov chain Monte Carlo simulation in the BEAST package v.1.7. The analysis used uncorrelated lognormal relaxed molecular clocks and a constant-population coalescent process prior over the phylogenies was selected. The HKY substitution model with a rate variation and a proportion of invariant sites (G+I) were used.

Technical Appendix Table. Authors and originating and submitting laboratories of the sequences used for phylogenetic analysis in this study from the Global Initiative on Sharing All Influenza Data (GISAID) EpiFlu database*

Segment, ID no.	Country of origin	Subtype	Collection date (YYYY-Mon-DD)	Isolate name	Originating and submitting laboratory	Authors
HA, EPI548493 NA, EPI548495	Japan	H5N8	2014-Nov-18	A/duck/Chiba/26-372-61/2014	National Institute of Animal Health	Takehiko Saito
HA, EPI548485, NA, EPI548487	Japan	H5N8	2014-Nov -18	A/duck/Chiba/26-372-48/2014	National Institute of Animal Health	Takehiko Saito
HA, EPI548623, NA, EPI548626	Netherlands	H5N8	2014-Nov -15	A/chicken/Netherlands/14015531/2014	Central Veterinary Institute	Rene Heutink; Frank Harders, Sylvia Verschuren-Pritz, , Alex Bossers, Guus Bouwstra Koch, Rene Ruth
HA, EPI547678, NA, EPI547683	Netherlands	H5N8	2014-Nov -14	A/chicken/Netherlands/14015526/2014	Central Veterinary Institute	Heutink, Frank Harders, Sylvia Verschuren-Pritz, Alex Bossers, Guus Koch, Ruth Bouwstra,
HA, EPI544756, NA, EPI544759	Germany	H5N8	2014-Nov -04	A/turkey/Germany/MV-R2472/2014	Friedrich-Loeffler-Institut	Elke Starick
HA, EPI517161, NA, EPI517163	Japan	H5N8	2014-Apr-13	A/chicken/Kumamoto/1-7-2014	National Institute of Animal Health, National Agriculture and Food Research Organization	K. Kanehira, N. Takemae, Y. Uchida, R. Tunekuni, H. Hikono, T. Saito
HA, EPI543010, NA, EPI543012	China	H5N8	2014-Jan-22	A/duck/Beijing/CT01/2014	Institute of Microbiology, Chinese Academy of Sciences	Di Liu
HA, EPI543002, NA, EPI543004	China	H5N8	2014-Jan-20	A/duck/Beijing/FS01/2014	Institute of Microbiology, Chinese Academy of Sciences	Di Liu
HA, EPI530054, NA, EPI530056	China	H5N6	2014-Jan-10	A/duck/Jiangxi/95/2014	BGI Shenzhen	Xu Bing, Zhang Tao, Li Xiaowen,
HA, EPI507673, NA, EPI507675	China	H5N8	2013-Nov-18	A/mallard duck/Shanghai/SH-9/2013	Institute of Military Veterinary, Academy of Military Medical Sciences, Institute of Laboratory Animal Sciences, Chinese Academy	S. Fan, X. Gao, Y. Ying, J. Guo, W. Sun, T. Wang, Z. Ren, Z. Yu, Y. Li,Y. Zhao, S. Yang, Y. Gao, X. Xia
HA, EPI542617, NA, EPI542627	China	H5N8	2013-Nov-10	A/duck/Beijing/FS01/2013	Institute of Microbiology, Chinese Academy of Sciences	Di Liu
NA, EPI339175	Germany	H6N8	2010-unknown-unknown	A/mallard/Germany-BY/R1353/2010	Friedrich-Loeffler-Institut	Elke Starick

*http://platform.gisaid.org/epi3/frontend#5412a6
